# Supplementary material for: Elevation, disturbance, and forest type drive the occurrence of a specialist arboreal folivore
Source: PLoS One. 2022 Apr 13;17(4):e0265963. doi: 10.1371/journal.pone.0265963 (PMC9007346; doi:10.1371/journal.pone.0265963)
Supplement: S1 Table — A) continuous variables, B) dominant tree species and C) Pearson correlations for the climate and elevation variables. (DOCX) [file pone.0265963.s001.docx]

**Table S1. Descriptive information for the covariates for all sites. A) continuous variables, B) dominant tree species and C) Pearson correlations for the climate and elevation variables.**

**(A)**

| Variable | Min | Max | Median | Mean | SD |
| --- | --- | --- | --- | --- | --- |
| Aspect (Degrees) | 2.3 | 359.8 | 188.8 | 181.5 | 108.1 |
| Elevation (m ASL) | 295.4 | 1228.2 | 799.2 | 811.1 | 187.9 |
| Number Days Max Daily Temp > 30 | 15 | 121 | 51 | 54.2 | 23.3 |
| Number Days Max Daily Temp > 35 | 0 | 22 | 3 | 6.3 | 5.9 |
| No Days Min Daily Temp > 20 | 0 | 23 | 7 | 6.9 | 4.0 |
| Number of Hollow Bearing Trees | 0 | 28 | 2 | 4.6 | 5.7 |
| Slope (Degrees) | 1.49 | 34.5 | 10.6 | 12.2 | 7.1 |
| Topographic Wetness Index | 4.36 | 11.0 | 6.1 | 6.3 | 1.2 |

**(B) Dominant species**

| **Tree species** | **Number** | **Percentage** |
| --- | --- | --- |
| *E. delegatensis* | 17 | 10.5% |
| *E. nitens* | 6 | 3.7% |
| *E. regnans* | 123 | 76.4% |
| Mixed | 15 | 9.3% |

**(C) Correlation amongst the climate covariates and elevation.**

|  | Elevation (m ASL) | No Days Max Daily Temp > 35 | No Days Min Daily Temp > 20 |
| --- | --- | --- | --- |
| Elevation (m ASL) | 1.000 | -0.857 | -0.729 |
| Number Days Max Daily Temp > 35 | -0.857 | 1.000 | 0.737 |
| Number Days Min Daily Temp > 20 | -0.729 | 0.737 | 1.000 |
