# Supplementary material for: Elevation, disturbance, and forest type drive the occurrence of a specialist arboreal folivore
Source: PLoS One. 2022 Apr 13;17(4):e0265963. doi: 10.1371/journal.pone.0265963 (PMC9007346; doi:10.1371/journal.pone.0265963)
Supplement: S2 Table — (A) continuous variables, (B) forest age and (C) Pearson correlations for the climate and elevation variables. (DOCX) [file pone.0265963.s002.docx]

**Table S2. Descriptive information for the covariates for Mountain Ash sites. (A) continuous variables, (B) forest age and (C) Pearson correlations for the climate and elevation variables.**

**(A)**

| Variable | Min | Max | Median | Mean | SD |
| --- | --- | --- | --- | --- | --- |
| Aspect (Degrees) | 7.8 | 359.8 | 194.3 | 184.3 | 104.4 |
| Elevation (m ASL) | 295.4 | 1126.5 | 761.9 | 760.8 | 156.6 |
| Number Days Max Daily Temp > 35 | 0 | 22 | 4 | 7.3 | 5.9 |
| Number Days Min Daily Temp > 20 | 1 | 23 | 8 | 7.6 | 3.8 |
| Number of Hollow Bearing Trees | 0 | 28 | 3 | 4.6 | 5.7 |
| Slope (Degrees) | 1.5 | 34.5 | 10.5 | 12.1 | 7.1 |
| Topographic Wetness Index | 4.4 | 11.0 | 6.1 | 6.3 | 1.3 |

**(B) Forest Age:**

| Age Group | Number | Percentage |
| --- | --- | --- |
| Old Growth | 14 | 11.4% |
| 1926-1939 | 72 | 58.5% |
| 1960-1990 | 17 | 13.8% |
| 2009 | 20 | 16.3% |

**(C) Correlation amongst the climate covariates and elevation**

|  | Elevation (m ASL) | Number Days Max Daily Temp > 35 | Number Days Min Daily Temp > 20 |
| --- | --- | --- | --- |
| Elevation (m ASL) | 1.000 | -0.864 | -0.654 |
| Number Days Max Daily Temp > 35 | -0.864 | 1.000 | 0.700 |
| Number Days Min Daily Temp > 20 | -0.654 | 0.700 | 1.000 |
