# Supplementary material for: Elevation, disturbance, and forest type drive the occurrence of a specialist arboreal folivore
Source: PLoS One. 2022 Apr 13;17(4):e0265963. doi: 10.1371/journal.pone.0265963 (PMC9007346; doi:10.1371/journal.pone.0265963)
Supplement: S3 Table — The most parsimonious model is highlighted in bold (the simplest model within 2 ΔLOOIC units of the best fitting model). (DOCX) [file pone.0265963.s003.docx]

**Table S3. Model selection results for the 24 models considered for the hurdle and conditional abundance components of the hurdle Poisson model. The most parsimonious model is highlighted in bold (the simplest model within 2 ∆LOOIC units of the best fitting model).**

|  | ∆LOOIC | |
| --- | --- | --- |
| Model | Hurdle Component | Conditional Abundance Component |
| Intercept | 19.238 | 4.213 |
| ForestAge | 4.774 | 7.04 |
| CanVis | 14.344 | 1.713 |
| CanVis + ForestAge | 5.225 | 5.297 |
| NumHBT | 12.518 | 3.549 |
| NumHBT + ForestAge | **0.896** | 8.292 |
| NumHBT + CanVis | 8.072 | 3.593 |
| NumHBT + CanVis + ForestAge | 1.795 | 7.054 |
| DaysMaxTemp35 | 21.027 | 2.765 |
| DaysMaxTemp35 + ForestAge | 6.427 | 5.921 |
| DaysMaxTemp35 + CanVis | 15.685 | 2.945 |
| DaysMaxTemp35 + CanVis + ForestAge | 5.893 | 6.35 |
| DaysMaxTemp35 + NumHBT | 12.793 | 3.971 |
| DaysMaxTemp35 + NumHBT + ForestAge | 0 | 8.291 |
| DaysMaxTemp35 + NumHBT + CanVis | 7.159 | 4.076 |
| DaysMaxTemp35 + NumHBT + CanVis + ForestAge | 0.937 | 8.011 |
| DaysMinTemp20 | 18.997 | 3.585 |
| DaysMinTemp20 + ForestAge | 6.503 | 6.554 |
| DaysMinTemp20 + CanVis | 11.497 | 3.651 |
| DaysMinTemp20 + CanVis + ForestAge | 4.906 | 7.487 |
| DaysMinTemp20 + NumHBT | 11.423 | 4.091 |
| DaysMinTemp20 + NumHBT + ForestAge | 1.842 | 7.594 |
| DaysMinTemp20 + NumHBT + CanVis | 4.348 | 5.611 |
| DaysMinTemp20 + NumHBT + CanVis + ForestAge | 1.572 | 9.133 |
| Elevation | 21.2 | **0** |
| Elevation + ForestAge | 6.449 | 4.539 |
| Elevation + CanVis | 15.501 | 1.386 |
| Elevation + CanVis + ForestAge | 5.841 | 4.95 |
| Elevation + NumHBT | 12.411 | 2.531 |
| Elevation + NumHBT + ForestAge | 0.625 | 6.915 |
| Elevation + NumHBT + CanVis | 6.116 | 3.735 |
| Elevation + NumHBT + CanVis + ForestAge | 0.927 | 7.359 |
