# Supplementary material for: Elevation, disturbance, and forest type drive the occurrence of a specialist arboreal folivore
Source: PLoS One. 2022 Apr 13;17(4):e0265963. doi: 10.1371/journal.pone.0265963 (PMC9007346; doi:10.1371/journal.pone.0265963)
Supplement: Attachment — Submitted filename: Response_to_Reviewers_Feb_2022.docx [file pone.0265963.s005.docx]

Professor David Lindenmayer

Fenner School of Environment and Society

The Australian National University

Canberra, ACT, 2601

28 February 2022

**Re: Further revisions to PONE-D-21-30842R1: Elevation, disturbance, and forest type drive the occurrence of a specialist arboreal folivore**

Dear Dr Angela Marie White, Ph.D,

Thankyou for your correspondence of 24 February 2022. We have now completed a further set of revisions to our manuscript, entitled: **Elevation, disturbance, and forest type drive the occurrence of a specialist arboreal folivore that was submitted to PLOS One.**

We have carefully considered and then responded to the comments from Reviewer #3. The changes we have made to the paper are outlined in a point-by-point response in the remainder of this letter.

We are most grateful to Professor Ross Goldingay (who was Reviewer #3) for his insightful comments. Addressing his suggested changes has helped further strengthen the manuscript.

We trust that this letter and the associated revised manuscript will be received favourably and look forward to hearing from you in the near future.

Yours sincerely,

David Lindenmayer

(On behalf of all authors)

**COMMENTS FROM REVIEWER #3**

**Comments from Reviewer #3**

This is an interesting and worthwhile study. The authors have done a good job of revising the ms based on the reviews of previous referees. I think some further revision will enhance the quality and readability of this ms. Some new paragraphs seem redundant given other text that is presented. Elsewhere I found there is existing or new text that is not concise and should be deleted. In a few places I think some additional references could be cited to link with earlier research conducted on this species. These suggestions are documented below.

Detailed comments and corrections

The line numbers refer to the clean copy.

**Response from authors**

We thank Reviewer #3 (Professor Goldingay) for their support of our study and also for his insightful comments. We have further revised the manuscript to address the comments made by Professor Goldingay. These changes have further strengthened the paper and we are grateful for the opportunity to revise the manuscript.

***********************************

**Comments from Reviewer #3**

Abstract

L32-33. This sentence can be deleted ‘We excluded data collected in these forest types in further analysis.’

**Response from authors**

We have removed this sentence as suggested by Reviewer #3.

***********************************

**Comments from Reviewer #3**

Introduction

L69. Add Kavanagh & Lambert (1990) Aust Wildl Res 17:285.

**Response from authors**

We have added the citation as requested.

***********************************

**Comments from Reviewer #3**

L70. Add Kavanagh & Wheeler (2004). p.413-25. In ‘The Biology of Australian Possums and Gliders’. Add Lindenmayer, et al. (2004). Wildlife Research 31, 569.

**Response from authors**

We have added these citations as requested.

***********************************

**Comments from Reviewer #3**

Methods

L116. Glider surveys were conducted during Dec 2020 to May 2021. Why are temperature data described for 2019-20 and yet the temperature data used in an analysis was from 2015-19?

**Response from authors**

We had to use available temperature and other weather data in our modelling; data at the time of our field surveys are currently still not available.

***********************************

**Comments from Reviewer #3**

L139. Table S1 describes 3 not 2 temperature variables.

**Response from authors**

We have corrected this error.

***********************************

**Comments from Reviewer #3**

Is it meaningful to go to 2 decimal places with all of the variables? In Table S2 as well.

**Response from authors**

We have rounded these values to a single decimal place.

***********************************

**Comments from Reviewer #3**

Change ‘No’ to ‘No.’

**Response from authors**

We have changed No to “number” in the tables to make interpretation easier for readers.

***********************************

**Comments from Reviewer #3**

L141 & 144. It would be helpful to know explicitly the period over which the number of days were estimated to avoid confusion (i.e. is it 1 Jan 2015 to 31 Dec 2019? i.e. 5 years not 4).

**Response from authors**

It is the full calendar year for each year – this has now been clarified in the revised Methods section.

***********************************

**Comments from Reviewer #3**

L148. ‘that remain’ needs some qualification.

**Response from authors**

We have removed the term “that remain” from the revised manuscript.

***********************************

**Comments from Reviewer #3**

L171. It would be helpful to the reader if you stated the benefits of modelling species’ absence rather than presence.

**Response from authors**

We have modelled species absence in this case, but presence is the opposite side of the same coin. Essentially in the hurdle part of the modelling, the first stage of the work is to quantifying the factors that influence absence/presence as we have done here.

***********************************

**Comments from Reviewer #3**

L199. It’s not clear whether there is justification to build models with 4 covariates.

**Response from authors**

We have data from 123 sites from which to construct our models. The number of potential explanatory variables should be at least an order of magnitude less than the number of sites – and we have elected to construct models with a relatively small number of covariates that are ecologically meaningful.

***********************************

**Comments from Reviewer #3**

L201-3. Awkward sentence structure. Perhaps put variables in brackets or simplify to be referred to as temperature variables.

**Response from authors**

We have reworked the sentence (and broken it into two shorter sentences) so that it can be more readily understood by readers.

***********************************

**Comments from Reviewer #3**

L206-8. Do the levels in the variables need to be restated here?

**Response from authors**

We have removed the repetition regarding the age classes from this part of the paper.

***********************************

**Comments from Reviewer #3**

L209-10. Given the high correlation between elevation and the 2 temperature variables it is not clear why all 3 should be retained. Two appear redundant. One temp variable certainly is.

**Response from authors**

At the outset of the analysis, it was unclear which of the potential explanatory variables would be most important. On this basis, we implemented our analysis via four sets of models (each set with 8 models) – giving 32 models all up. Only one temperature variable was fit within any given set. This is carefully described in the revised version of the manuscript.

***********************************

**Comments from Reviewer #3**

L223-229. Delete. This seems to be covered more concisely at L201-3. Note at L223 ‘presence’ rather than ‘absence’ is used.

**Response from authors**

We have revised this section of the paper as requested.

***********************************

**Comments from Reviewer #3**

There was a high correlation between the 2 temperature variables so only one should be used.

**Response from authors**

As outlined above, at the outset of the analysis, it was unclear which of the potential explanatory variables would be most important. On this basis, we implemented our analysis via four sets of models (each set with 8 models) – giving 32 models all up. Only one temperature variable was fit within any given set. This is carefully described in the revised version of the manuscript.

***********************************

**Comments from Reviewer #3**

L229-236. Delete. This section seems redundant given it precedes the results section in which one could concisely state what is presented, and leave any discussion of the models to the discussion.

**Response from authors**

We have removed the text in this part of the manuscript.

***********************************

**Comments from Reviewer #3**

Results

L240-46. This could be written more concisely. Some text seems repetitious of what is stated elsewhere (e.g. confining analysis to only the mountain ash forest). Describe the greater glider findings first.

**Response from authors**

We have reworked the text to make the writing more concise.

***********************************

**Comments from Reviewer #3**

L251. Delete ‘As outlined above,’

**Response from authors**

We have deleted this text.

***********************************

**Comments from Reviewer #3**

Table S3. It would help if the models were arranged from lowest to highest ∆LOOIC for one column.

**Response from authors**

We trialled the process of re-ordering the models as requested by Reviewer #3. However, the way the models are presented reflects the sequential addition of covariates and increasing model complexity. To reorder the models by ∆LOOIC makes the sequence of model construction quite confusing and we believe that it is best to retain it the way it is now – and with the best fitting model highlighted in bold.

***********************************

**Comments from Reviewer #3**

L260-1. Fig 2b should be Fig 2c in the text. Unconditional abundance is shown in 2d-f which could be cited with each of a, b, c.

**Response from authors**

We have checked the labelling of the Figures and corrected them as appropriate.

***********************************

**Comments from Reviewer #3**

L272-281. Fig. 2 caption. There is redundancy in this caption. Delete ‘The first row of the figure shows’. This is not concise. It also seems to be at odds with the layout of the Fig panels which are labelled a-f.

**Response from authors**

We have revised the caption for Figure 2 as requested.

***********************************

**Comments from Reviewer #3**

Discussion

I think the discussion could be more concise.

**Response from authors**

We have now carefully checked and then revised the entire Discussion section so that it is more concisely written.

***********************************

**Comments from Reviewer #3**

I think the first section of the discussion could be subdivided further to focus the reader’s attention. Perhaps rather than the formal subheading used currently, name the key variable identified – e.g. Influence of … Then a subsequent subheading (around L334) could be the Influence of forest type.

**Response from authors**

This was a good suggestion from Reviewer #3 and we have restructured the opening sections of the Discussion as requested.

***********************************

**Comments from Reviewer #3**

L290-3. This sentence is redundant.

**Response from authors**

We have removed this sentence as requested.

***********************************

**Comments from Reviewer #3**

L298. Add Kavanagh & Wheeler (2004); Lindenmayer et al. (2004).

**Response from authors**

We have added these citations to the revised manuscript.

***********************************

**Comments from Reviewer #3**

L313-15. It needs to be made clear in the study area description that the younger stand ages may contain some old growth elements. Perhaps because stand replacing fires may be patchy.

**Response from authors**

This was an astute point and we have now included this extra detail in the revised Methods section of the paper.

***********************************

**Comments from Reviewer #3**

L317. There seems some repetition here.

**Response from authors**

We have removed the repetition from this part of the paper.

***********************************

**Comments from Reviewer #3**

L319-22. The point here about detection and forest age could be made more concise. I assume this point may be less relevant when you model prob of absence.

**Response from authors**

We have removed much of the text at the end of this section as suggested by Reviewer #3.

***********************************

**Comments from Reviewer #3**

L326. I think it worth also citing reference 18 which first identified an influence of elevation on- greater glider occupancy or decline.

**Response from authors**

We have added this citation in this part of the paper.

***********************************

**Comments from Reviewer #3**

L331. Or the climate model predicted temperature less well than elevation.

**Response from authors**

This is a good point and we have added this caveat to the revised text.

***********************************

**Comments from Reviewer #3**

L334. High not higher.

**Response from authors**

We have corrected this typographical error.

***********************************

**Comments from Reviewer #3**

L337. I think Kavanagh & Stanton (1998) (Aust Zool 30:449) should be cited here in relation to paucity in alpine ash and at the highest elevations.

**Response from authors**

We have added this citation in this part of the paper.

***********************************

**Comments from Reviewer #3**

L338. This sentence could be deleted.

**Response from authors**

We have deleted this sentence as recommended.

***********************************

**Comments from Reviewer #3**

L347-9. This sentence should be placed at the end of the section.

The following reference should also be cited. Salmona, J., Dixon, K.M., and Banks, S. C. (2018). The effects of fire history on hollow-bearing tree abundance in montane and subalpine eucalypt forests in southeastern Australia. Forest Ecology and Management 428, 93–103.

**Response from authors**

We have moved the sentence and added the citation as recommended.

***********************************

**Comments from Reviewer #3**

Ross Goldingay

**Response from authors**

We again thank Professor Goldingay for his most helpful and insightful comments.

***********************************
